# Targeted screening of multiple anti-inflammatory components from *Chrysanthemi indici* Flos by ligand fishing with affinity UF-LC/MS

**DOI:** 10.3389/fphar.2024.1272087

**Published:** 2024-04-16

**Authors:** Chuanqi Huang, Xin Xiong, Dan Zhang, Qingfeng Ruan, Jie Jiang, Fuqian Wang, Guilin Chen, Lu Cheng

**Affiliations:** ^1^ Department of Pharmacy, Wuhan Hospital of Traditional Chinese and Western Medicine, Wuhan, China; ^2^ Key Laboratory of Plant Germplasm Enhancement and Specialty Agriculture, Wuhan Botanical Garden, Chinese Academy of Sciences, Wuhan, China

**Keywords:** *Chrysanthemi indici* Flos, inflammation, skin diseases, COX-2, HAase, UF-LC/MS

## Abstract

*Chrysanthemi indic* Flos (CIF) has been commonly consumed for the treatment of inflammation and related skin diseases. However, the potential bioactive components responsible for its anti-inflammatory and sensitive skin (SS) improvement activities, and the correlated mechanisms of action still remain unknown. In this work, it was firstly found that the CIF extract (CIFE) displayed arrestive free radical scavenging activity on DPPH and ABTS radicals, with no significant difference with positive control Trolox (*p* > 0.05). Then, compared to the negative group, CIFE markedly decreased the productions of the pro-inflammatory cytokines (IL-1β, IL-6, PEG2, TNF-α, IFN-γ, NO) in LPS induced RAW264.7 cells in a dose-dependent manner (*p* < 0.01). Besides, CIFE strongly inhibited the COX-2 and hyaluronidase (HAase) with the IC50 values of 1.06 ± 0.01 μg/mL and 12.22 ± 0.39 μg/mL, indicating higher inhibitory effect than positive control of aspirin of 6.33 ± 0.05 μg/mL (*p* < 0.01), and comparable inhibitory effect with indometacin of 0.60 ± 0.03 μg/mL, and ascorbic acid of 11.03 ± 0.41 μg/mL (*p* > 0.05), respectively. Furthermore, kinetic assays with Lineweaver-Burk plot (Michaelis Menten equation) suggested that CIFE reversibly inhibited the COX-2 and HAase, with a mixed characteristics of competitive and non-competitive inhibition. Thereafter, multi-target affinity ultrafiltration liquid chromatography-mass spectrometry (UF-LC/MS) method was employed to fast fish out the potential COX-2 and HAase in CIFE. Herein, 13 components showed various affinity binding degrees to the COX-2 and HAase, while those components with relative binding affinity (RBA) value higher than 3.0, such as linarin and chlorogenic acid isomers, were deemed to be the most bioactive components for the anti-inflammatory and SS improvement activities of CIFE. Finally, the interaction mechanism, including binding energy, inhibition constant, docking sites, and the key amino acids involved in hydrogen bonds between the potential ligands and COX-2/HAase were simulated and confirmed with the molecule docking analysis. In summary, this study showcased the prominent anti-inflammatory and SS improvement activities of CIF, which would provide further insights on this functional medicinal plant to be a natural anti-SS remedy.

## 1 Introduction

Due to increasingly serious environmental pollution, the development of diversified cosmetics, and the increasing awareness of skin health, concerns about skin sensitivity are gradually increasing. Sensitive skin (SS), also known as Sensitive Skin Syndrome, is a skin condition characterized by high reactivity and poor tolerance, making it prone to allergies and impaired skin barrier function (Fan et al., 2016). SS mainly occurs on the face, with subjective symptoms such as burning, redness, tingling, pruritus, and may be accompanied by objective signs such as erythema, telangiectasia, desquamation, and even allergic reactions such as redness, swelling, and rash in severe cases (Berardesca et al., 2013). At present, antihistamines, glucocorticoids and antibiotics are the common means for skin diseases, with the advantage of quick response, but their side effects cannot be ignored. While the herbal medicines come from natural sources and have the advantages of low irritation and multiple targets (Zhang et al., 2020). In addition, the pursuit of green and natural cosmetics has made the research on natural soothing active substances a popular direction.

*Chrysanthemi indici* Flos (CIF), the dried flower head of *Chrysanthemum indicum* L. in the Asteraceae family, is widely distributed in China and has been commonly used for medicine and tea consumption. Currently, it is included in the Chinese Pharmacopoeia, and also in the list of Chinese herbal medicines for both food and medicine by the Ministry of Health of China (Shao et al., 2020). CIF contains various chemical components, such as flavonoids, terpenoids, phenylpropanoids, phenolic acids and polysaccharides, and has exhibited remarkable anti-inflammatory activities (Chen J. et al., 2019). For example, the CIF ethanol extract alleviated the skin irritation in mice caused by long-term exposure to 12-oxytetradecanol 13-acetate (Lee et al., 2009). Also, the CIF extract retarded the development of skin diseases, such as specific dermatitis and ear swelling in animal models (Lee et al., 2009; Park et al., 2012), and this therapeutic effect was closely related to the reduction of immunoglobulin E (IgE), interferon-γ (IFN-γ) and interleukin-4 (IL-4) levels in serum (Park et al., 2012). Meanwhile, the sesquiterpenoids of kikkanols A, B, C, and the flavonoid glycosides eriodictyol 7-*O*-β-D-glucopyranosiduronic acid, acaciin and luteolin 7-*O*-β-D-glucopyranoside, effectively reduced the expressions of IL-4 and IL-13 in dinitrochlorobenzene treated HaCaT cells and human keratinocytes (Matsuda et al., 2002). Furthermore, CIF inhibited the production of lipopolysaccharide-induced inflammatory factors by blocking the nuclear transcription factor (NF-κB) and mitogen activated protein kinase signal pathways in RAW 264.7 cells, thus playing an immunomodulatory role (Cheon et al., 2009).

Generally speaking, the high responsiveness and low tolerance of SS to external stimuli make it highly susceptible to skin irritation and allergy. Skin irritation triggers irritant contact dermatitis (ICD) through non immune mechanisms; skin allergy belongs to the immune response, and the most common skin disease is allergic contact dermatitis (ACD) (Fan et al., 2016). Considering that the both allergic reactions above cause damages to the skin barrier, trigger inflammation, and release inflammatory factors, inhibiting the production of inflammatory factors will effectively alleviate SS. Hyaluronic acid (HA) is a high molecular weight polysaccharide with high hydrophilicity. It maintains skin moisture and elasticity, promotes wound healing, regulates blood vessel formation (Voigt and Driver, 2012), and also participates in regulating inflammation and allergic reactions, which hence is closely related to SS (Marinho et al., 2021). Hyaluronidase (HAase) is a specific lyase of HA, its high activity will lead to the degradation of HA, thus losing these functions. Therefore, inhibiting HAase will alleviate allergic reactions by maintaining the function of HA. In addition, in the process of inflammation, arachidonic acid generates prostaglandins (PGs) under the action of cyclooxygenase 2 (COX-2). PGs is a class of strong inflammation-causing and pain-causing medium, especially PG2 (Yao and Narumiya, 2019). Our previous studies revealed that Qingxue jiedu Formulation with CIF as one of the main components improved the IL-12, TNF-α, IL-4, IL-6, IgE and IFN-γ levels in DNFB-induced atopic dermatitis mice. The mechanism was closely related to activation of STAT3, MAPK and NF-κB signaling pathways (Xiong et al., 2021). However, whether HAase and COX-2 were related to its ameliorating effect on SS, as well as the corresponding potential active components and mechanism of action still needed to be further studied. Base on the intermolecular affinity principle, the ultrafiltration liquid chromatography-mass spectrometry (UF-LC/MS) combines the LC-MS technology and ultrafiltration, which could be applied to quickly and conveniently identify potential ligands that bind to protein receptors and aims to achieve rapid screening of bioactive components or lead compounds from natural products (Chen et al., 2018). In this study, the two enzymes of HAase and COX-2 were targeted to screen the potential ligands by UF-LC/MS method, and then the enzyme kinetics experiments were further employed to determined the inhibitory types of CIF and potential ligands on enzymes. In this regard, this study will provide comprehensive elucidation for the screening of bioactive anti-SS components in CIF and their mechanisms of action.

## 2 Materials and methods

### 2.1 Plant materials

The dry *Chrysanthemi indici* Flos (batch number: 190,513) were obtained from Bozhou Zhongqiang pharmaceutical company Ltd. The voucher specimen was kindly authenticated by Professor Hezhen Wu, a senior TCM identification expert, which thereafter was deposited in the Chinese medicine herbarium of Wuhan No.1 Hospital (No. 20191216).

### 2.2 Sample preparation

For the preparation of *Chrysanthemi indici* Flos extract (CIFE), 200 g dry flower was soaked in pure water overnight, and then boiled for 1 h. Thereafter, the decoction was filtrated with medical gauze (12 layers). The above operation was repeated twice. The three decoctions were combined and concentrated under vacuum to obtain the brown syrup, which was finally freeze-dried to provide the dry powder of CIFE.

### 2.3 Antioxidant assays

The DPPH and ABTS free radicals scavenging activities of the CIFE were carried out in 96-well microliter plate on basis of the previous methods (Chen G. L. et al., 2019). The ascorbic acid and Trolox (3–300 μg/mL) were served as the positive controls, and all the assays were performed in triplicate. Finally, the antioxidant capacities of CIFE were displayed to be the half inhibitory concentration (IC50) values in DPPH and ABTS assays.

### 2.4 Anti-inflammatory assays

The RAW264.7 macrophage cell was obtained from American Type Culture Collection (ATCC). The cell was then kept in RPMI 1640 medium with 1% penicillin-streptomycin, and 10% FBS in a humidified incubator with 5% CO_2_. Later, the nitrite concentration and the inflammatory cytokines were determined according to the previous studies (Xiong et al., 2021; Zhang et al., 2022) with some modifications. In special, the NO concentration was detected with the Griess reagent, and the cytokines including the IL-1β, IL-6, PGE2, tumor necrosis factor α (TNF-α), and interferon γ (IFN-γ) in RAW 264.7 cell after CIFE treatment were detected with the commercial enzyme-linked immunosorbent assay (ELISA) kits (eBioscience, United States). Cells without LPS stimulation was used as normal control (Nor). The aspirin (10 μg/mL) was indicated to be the positive control, and all the detection was carried out three times.

### 2.5 COX-2 inhibition assay

The inhibition of CIFE on the enzyme COX-2 was performed with the commercial COX-2 inhibitor kits according to the previous work (Chen et al., 2021). Briefly, the detailed operation process was referred to the manufacturer’s instructions in dull or low light environment. Therein, the aspirin/indometacin and Tris-HCl buffer (pH 7.8) served as the positive and blank controls, respectively. Each sample was tested in triplicate, and the IC_50_ value (mean ± standard deviation, M ± SD) was used to evaluate the anti-inflammatory activity of CIFE on COX-2.

### 2.6 HAase inhibition assay

The inhibition assay of CIFE against HAase was measured according to a previous method (Srivastav et al., 2010) with some modifications. Briefly, 10 μL of sample solution and 50 μL of bovine HAase (Sigma-Aldrich, United States) were mixed into the eaction tube, which was incubated in water bath at 37°C for 20 min. Then, 10 μL of CaCl_2_ (12.5 mM) was added, and the reaction solution was kept in water bath for another 20 min at 37°C. After that, 250 μL of sodium hyaluronate (1.5 mg/mL, pH3.5) was added, mixed and then bathed at 37°C for further 40 min. Later, 100 μL of NaOH (0.4 M) and K_2_B_4_O_7_ (0.04 M) each were added into the above reaction solution, which was kept in boiling water for 3 min. After cooled down to room temperature, 20 μL of reaction solution was transferred to the 96 microplate, and the optical density (OD) value at 585 nm was determined by the Tecan multifunctional microplate reader (Infinite M200 PRO, Switzerland). The ascorbic acid was used as the positive control (Okorukwu and Vercruysse, 2003), and the OD value was employed to calculate the inhibition rate of hyaluronase activity, which was presented as follows:

$$\text{Inhibition }\left(\%\right)=\frac{\text{ODc}-\text{ODs}}{\text{ODc}}\times 100$$

where, ODc: absorbance of the control, ODs: optical density of the tested sample. Meanwhile, 10 μL of ultra-pure water was used as blank control. Each sample was performed three times.

### 2.7 Affinity ultrafiltration screening

The potential anti-inflammatory ligands in *Chrysanthemi indici* Flos targeting COX-2 and HAase were fished out by multiple affinity ultrafiltration screening. In this regard, the UF-LC/MS process was implemented based on the earlier studies (Chen et al., 2021; Xu et al., 2022) with some modifications. Briefly, the CIFE was accurately weighed and then dissolved in Tris-HCl buffer (pH 7.8, for COX-2) or PBS buffer (pH 5.4, for HAase) with the final concentration of 2.0 mg/mL. The usages of COX-2 and HAase were 5 U and 30 U, which corresponded with the 30 and 10 KD cutoff ultrafiltration tubes (0.5 mL, Millipore), respectively. The enzymes denaturated in boiling water for 10 min was served to be negative control, and the above screening process was repeated in triplicate. Finally, the eluents were conbined and freeze-dried for the further LC-MS analysis.

### 2.8 LC-MS analysis

The qualitative and quantitative analysis of the potential ligands in CIFE were carried out with the Thermo Accela 600 series HPLC system, which was connected with a TSQ Quantum Access MAX mass spectrometer (Thermo Fisher Scientific, United States). For the HPLC analysis, a Waters Sunfire RP-C18 column (4.6 mm × 150 mm, 5 μm) was employed. 0.1% formic acid—H_2_O (0.1% FA-H_2_O, A) and acetonitrile (ACN, B) were used as the mobile phases. The HPLC elution sequences were: 0–2 min, 5%B; 2–30 min, 5%–45% B; 30–35 min, 45%–95% B; 35–40 min, 95%–5%. The flow rate, injection volume, column temperature and UV detection wavelength were set as 800 μL/min, 10 μL, 30°C and 320 nm, respectively. For the ESI-MS/MS analysis, the optimized MS instrument parameters in negative ion mode were: capillary temperature, 350°C; vaporizer temperature, 300°C; spray voltage, 3.0 KV; sheath gas (N_2_), 40 psi; mass range, m/z 100–1000. The full-scan and data-dependent mode was employed to obtain the mass spectrum data, which was further analyzed with the Thermo Xcalibur ChemStation.

### 2.9 Molecular docking analysis

The molecular interaction simulation between COX-2 (PDB: 1PXX), HAase (PDB: 1FCV) and their potential ligands was illustrated with the computer aided drug design software of AutoDock Tools (V 1.5.6) and Discovery Studio (V 4.1) based on previous studies with some modifications (Chen et al., 2021; Xu et al., 2022). Briefly, the 3D structures of target enzymes (COX-2, HAase) and ligands with the lowest energy were firstly obtained from the RSCB Protein Database (www.rcsb.org) and ChemBio3D Ultra (V 14.0), respectively. Then, the pre-treatment processes of dewatering, hydrogenation, point-charge, and protonation confirmation were implemented with the AutoDock Tools. After that, a grid box with dimensions of 60 × 60 × 60 Å was centered on the active site of target enzyme, accompanied by the coordinates of *x*_30.415, *y*_16.244, *z*_16.922 for COX-2, and *x*_4.447, *y*_29.902, *z*_-9.110 for HAase, respectively. Later, the molecular docking analysis was performed with 2.5 × 10^6^ energy assessments, 50 independent genetic algorithms runs and other default parameters. Finally, the visual 3D geometry conformations with the lowest docking energy were obtained with the AutoDock Tools and Discovery Studio.

### 2.10 Kinetic assays

The apparent kinetic parameters of CIFE on COX-2 and HAse were determined, including Michaelis constant (Km) and maximum reaction velocity (Vm), according to the methods of the COX-2 and HAase inhibition assays above. Then, based on the Michaelis Menten equation, the Lineweaver Burk double reciprocal equation was used to plot and analyze the effects of different concentrations of CIFE and substrates (hematin, sodium hyaluronate) on the reaction rate (Li et al., 2022). The reversibility of CIFE on COX-2 and HAase, as well as the types of reversible inhibition reactions, were determined. Usually, the relationship between the enzyme reaction velocity (V) and the substrate concentration (S) can be expressed by the Michaelis equation in the single substrate enzyme catalysis. Thereinto, the double reciprocal mapping method was used for parameter estimation, namely, the reciprocal of the Michaelis equation:

$$\frac{1}{V}=\frac{1}{\text{Vm}}+\frac{\text{Km}}{\text{Vm}}\times \frac{1}{S}$$

where the Lineweaver-Burk plot was obtained by plotting 1/Vm against 1/S through experiments.

### 2.11 Statistical analysis

All tests were carried out with three parallel samples, and the results were expressed as M ± SD. The SPSS Statistics 26.0 software was used for the one-way ANOVA statistical analysis, and *p* < 0.05 indicated significant differences between groups. The Origin Pro 8 software was employed for drawing.

## 3 Results

### 3.1 Antioxidant capacities of CIFE

The antioxidant capacities of CIFE were currently assessed with the electron transfer based free radical scavenging activities, including the DPPH and ABTS assays. As shown in Table 1, the CIFE exhibited strong scavenging potential on DPPH and ABTS radicals compared with the positive controls of ascorbic acid and Trolox. In special, CIFE displayed no significant difference with Trolox in scavenging DPPH and ABTS radicals (*p* > 0.05), with the IC50 values of 103.47 ± 4.83 μg/mL and 56.25 ± 1.82 μg/mL, respectively.

**TABLE 1:** The scavenging and enzyme inhibitory activities of CIFE on free radicals, COX-2 and HAase.

	IC50 (µg/mL)			
	DPPH	ABTS	COX-2	HAase
CIFE	103.47 ± 4.83^a^	56.25 ± 1.82^a^	1.06 ± 0.01^b,c^	12.22 ± 0.39^d^
Ascorbic acid	67.35 ± 1.60	43.62 ± 1.67	-	11.03 ± 0.41
Trolox	96.26 ± 4.12	53.73 ± 1.95	-	-
Aspirin	-	-	6.33 ± 0.05	-
Indometacin	-	-	0.60 ± 0.03	-

CIFE, *chrysanthemi indici* flos extract; DPPH, 2,2-diphenyl-1-picrylhydrazyl; ABTS, 2,20-azinobis-(3-ethylbenzthiazoline-6-sulfonic acid); HAase, hyaluronidase. M ± SD, value was calculated with three independent IC50 determination of each sample in parallel. a, *p* > 0.05, compared with positive control Trolox; b, *p* < 0.01, compared with positive control aspirin; c, *p* > 0.05, compared with positive control indometacin; d, *p* > 0.05, compared with positive control ascorbic acid.

### 3.2 Inhibitory effects of CIFE on the inflammatory cytokines in RAW 264.7 cells

**FIGURE 1:**
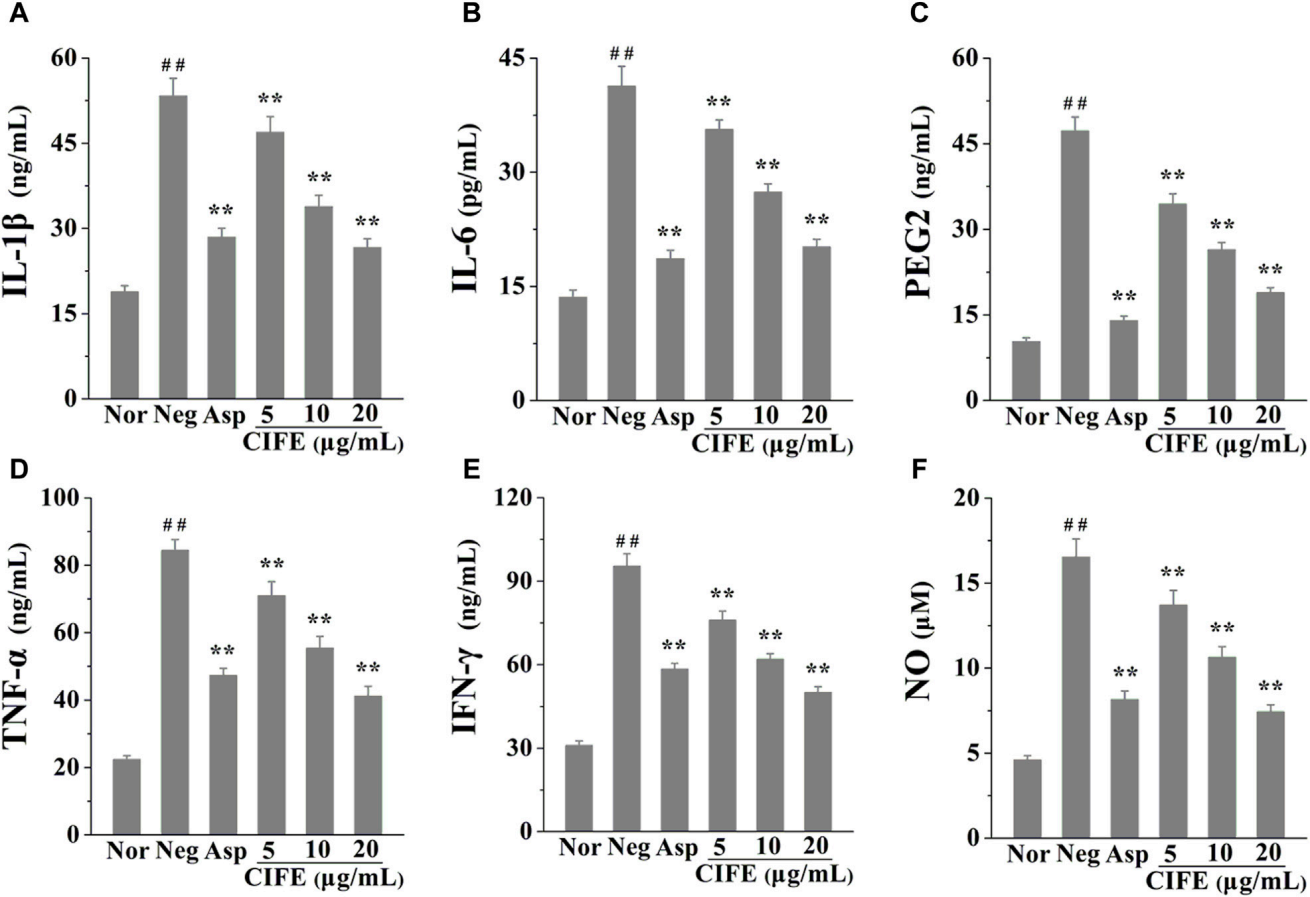
Regulation of CIFE on inflammatory cytokines of IL-1β **(A)**, IL-6 **(B)**, PEG2 **(C)**, TNF-α **(D)**, IFN-γ **(E)**, and NO **(F)** in RAW 264.7 cells. ^##^ *p* < 0.01, compared with normal control group (Nor); ^**^ *p* < 0.01, compared with negative control group (Neg).

The anti-inflammatory effect of CIFE was assessed by detecting the inflammatory mediators levels in LPS stimulated RAW 264.7 cells. As shown in Figures 1A–F, compared with the normal control group (Nor), the levels of pro-inflammatory cytokines, including IL-1β, IL-6, PEG2, TNF-α, IFN-γ and NO, were significantly increased in negative control group (Neg, *p* < 0.01). Nevertheless, the levels the aforementioned cytokines were markedly reversed after the intervention of CIFE in a dose-dependent manner, compared to the Neg group (*p* < 0.01). Especially, CIFE at 20 μg/mL exhibited comparable and/or slightly stronger inhibition effect on the production of above cytokines with the positive control of aspirin at 10 μg/mL (*p* > 0.05), thus further verifying its attractive anti-inflammatory potential.

### 3.3 Enzyme inhibitory activities of CIFE against the COX-2 and HAase *in vitro*

In view of the stimulating effect of inflammation in the process of SS, particularly the key role of COX-2 during inflammatory response and the side effect of HAase on skin repair, the inhibition of CIFE against the enzymes of COX-2 and HAase were further determined. The results in Table 1 suggested that the CIFE exerted lower IC50 value of 1.06 ± 0.01 μg/mL on COX-2 than the positive control of aspirin of 6.33 ± 0.05 μg/mL (*p* < 0.01), and comparable inhibitory effect with indometacin of 0.60 ± 0.03 μg/mL (*p* > 0.05). Also, the CIFE displayed comparable inhibitory effect with the positive control ascorbic acid against HAase with the IC50 values of 12.22 ± 0.39 μg/mL and 11.03 ± 0.41 μg/mL (*p* > 0.05), respectively.

### 3.4 Inhibition type analysis

**FIGURE 2:**
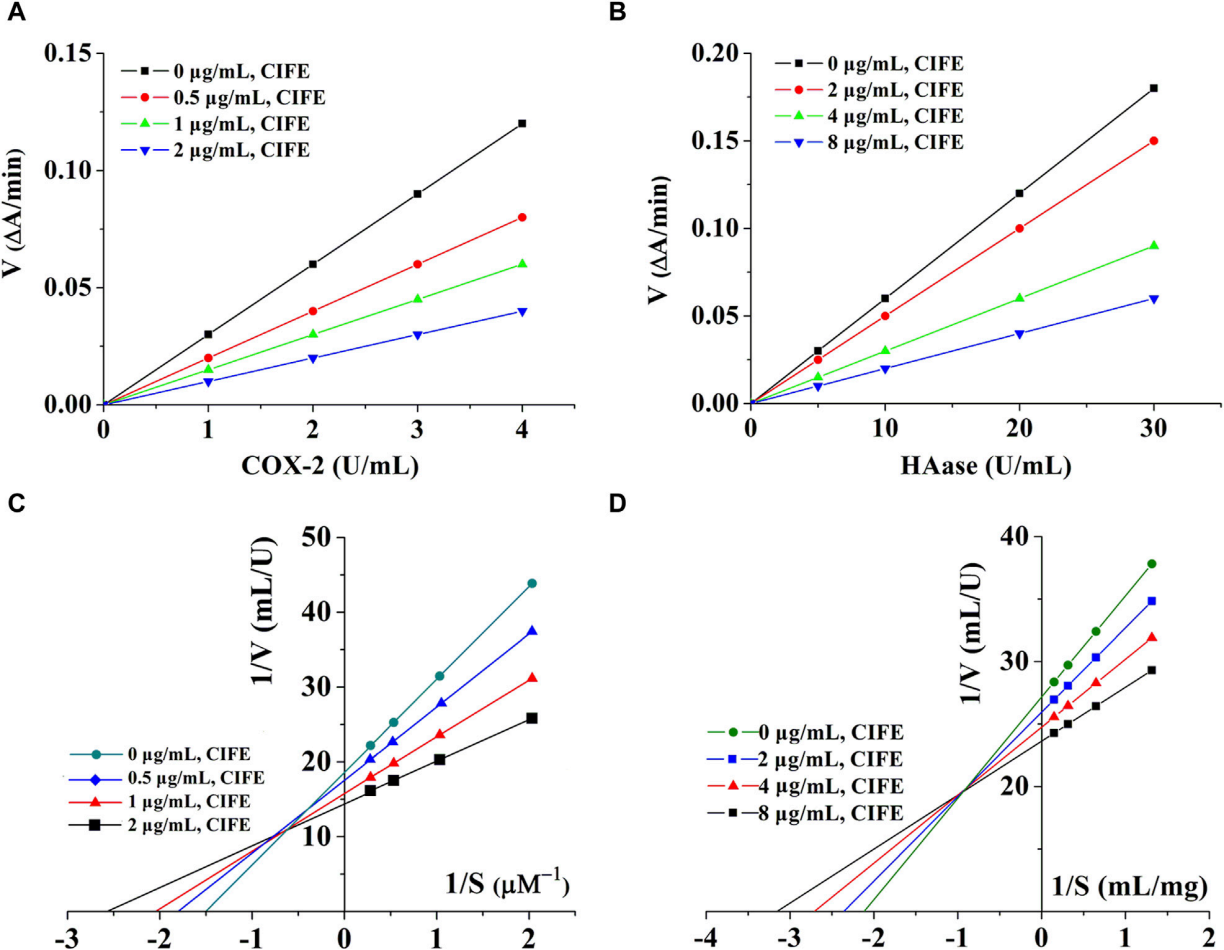
Kinetics curves of CIFE on COX-2 **(A)**, Haase **(B)**, and Lineweaver-burk curves of CIFE against COX-2 **(C)**, Haase **(D)**. ΔA, the variations in absorbance at the beginning and end of the reaction. V, reaction velocity. S, substrate.

By changing the concentration of CIFE, a set of straight lines can be obtained by plotting enzymes of different concentrations. As shown in Figures 2A,B, all lines were linear and passed through the origin. In addition, the slopes of the lines decreased with the increase of CIFE concentration, indicating that CIFE did not change the number of enzymes, but decreased the activity of enzymes. Therefore, the inhibitory effects of CIFE on COX-2 and HAase were reversible.

In order to clarify the reversible inhibition type of CIFE on COX-2 and HAase, the inhibition kinetics of CIFE on COX-2 and HAase were studied by monitoring the changes of Km (Mi constant) and Vm (maximum reaction rate) in Lineweaver-Burk diagram. Thereinto, the slope was the ratio of Km to Vm, and the y-intercept represented the value of 1/Vm. As shown in Figures 2C,D, the Lineweaver-Burk curves of CIFE on COX-2 and HAase were a set of straight lines that intersected in the second quadrant. With the increase of CIFE concentration in the system, Vm and Km gradually decreases and increases, respectively, indicating the mixed inhibition characteristics of competition and non-competition.

### 3.5 Screening for potential COX-2 and HAase ligands in CIFE

**FIGURE 3:**
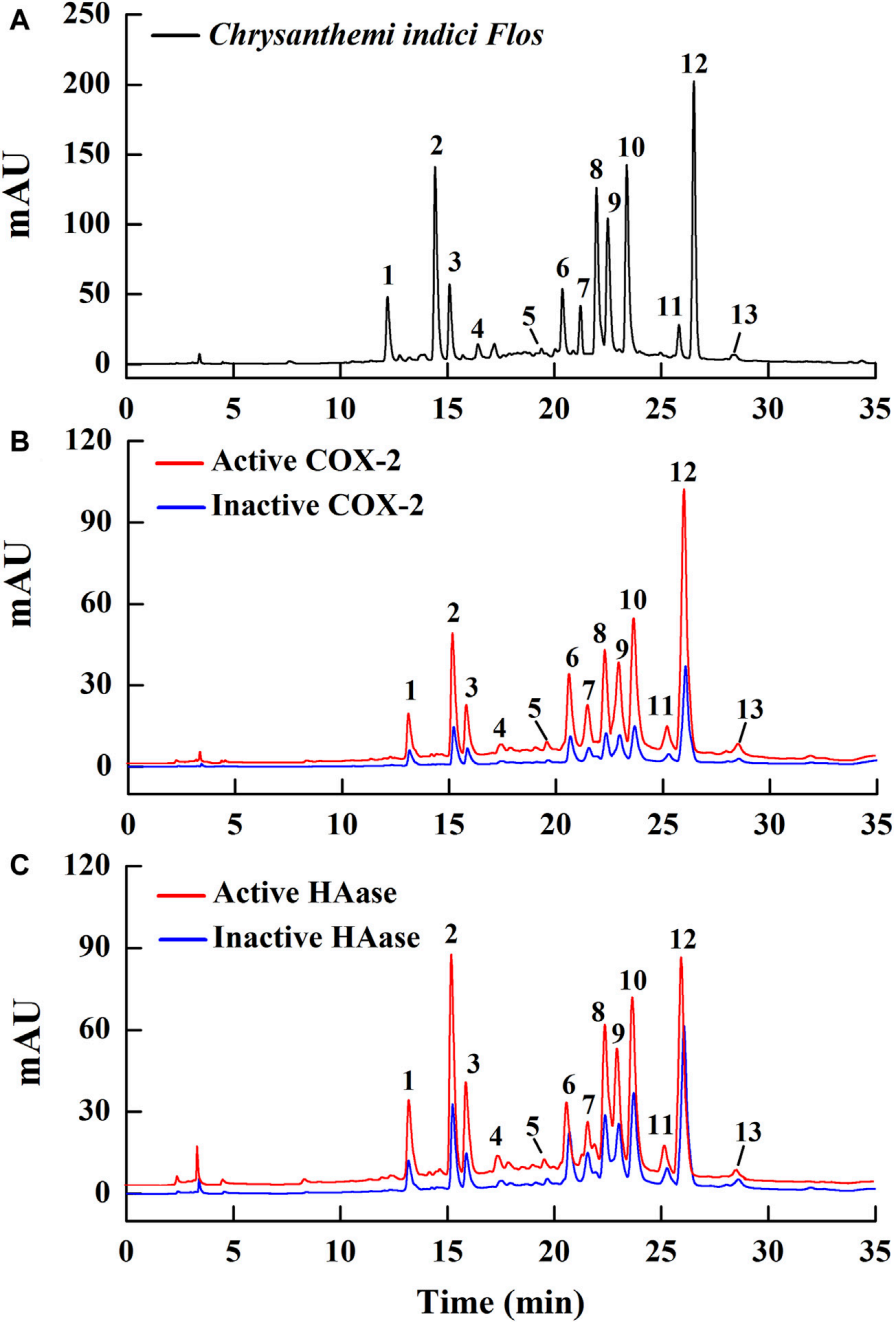
The HPLC chromatogram profiles of the potential ligands in *Chrysanthemi indici* Flos targeting COX-2 and HAase after affinity ultrafiltration. The black line indicates the chemical chromatogram of CIFE obtained at 320 nm **(A)**; The red and blue lines display the CIFE with the activated and incativated COX-2 **(B)** and HAase **(C)**, respectively.

Within herbal medicines, commonly multiple bioactive compounds contribute together to their pharmacological activities. In current work, the affinity ultrafiltration liquid chromatography and mass spectrometry (UF-LC/MS) method was applied to fast fish out the potential COX-2 and HAase ligands in CIFE, which is an efficient technology for fast discovering small molecule ligands with strong affinities to target proteins from complex natural products mixtures (Chen et al., 2018). After incubation with the active or inactive COX-2/HAase, the potential ligands were released from CIFE and then detected with HPLC. As shown in Figures 3A–C, 13 components showed various affinity binding degrees to the COX-2 and HAase.

Generally, the chromatograms of 13 components targeted to active and inactive COX-2/HAase displayed obvious differences in the peak areas. In order to quantify the binding strengths between these components and target enzymes, the relative binding affinity (RBA) based on the changes of the peak areas before and after incubation was employed and assessed via the equation:

$$\text{RBA}=A_{\text{ac}}/A_{\text{inac}}$$

Thereof, A_ac_ and A_inac_ indicate the peak areas of 13 components in CIFE interacting with active and inactive COX-2/HAase, respectively. Consequently, the RBA values of those 13 components in CIFE exhibiting specific binding strengths to COX-2 and HAase were calculated and displayed in Table 2. As for COX-2, peak 12 exerted the highest binding affinities with RBA value of 3.43, followed by peaks 8, 10, 9, 3, 2, 1, 4, 7 with higher RBA values of 2.97, 2.94, 2.85, 2.75, 2.74, 2.63, 2.60, and 2.55, respectively. With regard to HAase, peaks 1, 2, 3, 8, 10, 9, 12, 7, 5, 4, 6 displayed stronger binding strengths with the RBA values of 5.47, 5.44, 5.36, 4.84, 3.62, 3.45, 3.03, 2.83, 2.81, 2.72, and 2.61, respectively.

**TABLE 2:** The potential COX-2 and HAase ligands fished out from *Chrysanthemi indici* Flos.

Peak no.	Rt (min)	[M-H]^-^	MS/MS fragments (m/z)	Identification	RBA		References
					COX-2	HAase	
1	12.20	353.0717	191, 173, 127	Neochlorogenic acid^a^	2.63	5.47	Tian et al. (2020)
2	14.43	353.0859	191, 173, 135	Chlorogenic acid^a^	2.74	5.44	Tian et al. (2020)
3	15.10	515.1160	515, 353, 191	1,3-Dicaffeoylquinic acid^b^	2.75	5.36	Shao et al. (2020)
4	16.42	609.1396	609, 301, 300	Rutin^a^	2.60	2.72	Tian et al. (2020)
5	19.39	447.4438	447, 285, 284	Luteoloside^b^	1.98	2.81	Chen J. et al. (2019)
6	20.37	449.1073	449, 287, 151	Astilbin^b^	2.47	2.61	Tian et al. (2020)
7	21.22	463.0777	463, 287, 175	Isoquercitrin^b^	2.55	2.83	Tian et al. (2020)
8	21.97	515.4883	515, 353, 335, 179, 173	Isochlorogenic acid B^a^	2.97	4.84	Tian et al. (2020)
9	22.49	515.4301	515, 353, 335, 179, 173	Isochlorogenic acid A^a^	2.85	3.45	Tian et al. (2020)
10	23.38	515.4681	515, 353, 335, 179, 173	Isochlorogenic acid C^a^	2.94	3.62	Tian et al. (2020)
11	25.82	445.0378	447, 269, 175, 113	Baicalin^b^	2.41	2.45	Tian et al. (2020)
12	26.52	637.1762	637, 591, 283, 268	Linarin^a^	3.43	3.03	Shao et al. (2020)
13	28.45	285.0369	285, 243, 217, 151	Luteolin^b^	1.60	1.44	Tian et al. (2020)

a, identified with standard chemicals; b, identified with the MS/MS, fragments of published data; Rt, retention time; RBA, relative binding affinity; HAase, hyaluronidase.

Generally speaking, compound with the RBA at the range of 1.5–2.0 was considered as weak ligand, moderate and strong binding affinities between 2.0–3.0, and higher than 3.0 (Munigunti et al., 2011), respectively. In this regard, on the one hand, those components, peculiarly with RBA value higher than 3.0, were deemed to be the most potential COX-2 and HAase ligands in CIFE. On the other hand, the diverse RBA values might be likely due to the competitive interactions between the aforementioned potential ligands and COX-2/HAase enzymes.

### 3.6 Structural identification of the HAase and COX-2 ligands by LC-MS/MS

In present work, the identification of 13 potential ligands targeting to HAase and COX-2 was carried out with the HPLC-UV/ESI-MS/MS analysis under the negative ion mode. Briefly, 13 components in CIFE were confirmed or tentatively identified by comparing the representative MS/MS fragments and their fragmentation pathways with the correlated standard chemicals and those reported in previous studies (Chen J. et al., 2019; Shao et al., 2020; Tian et al., 2020). The MS and MS/MS information of 13 potential HAase and COX-2 ligands, including the retention time (Rt), deprotonated molecular ion ([M-H]^-^) and the representative MS/MS fragments, were listed in Table 2 in detail.

### 3.7 Molecular docking analysis

**FIGURE 4:**
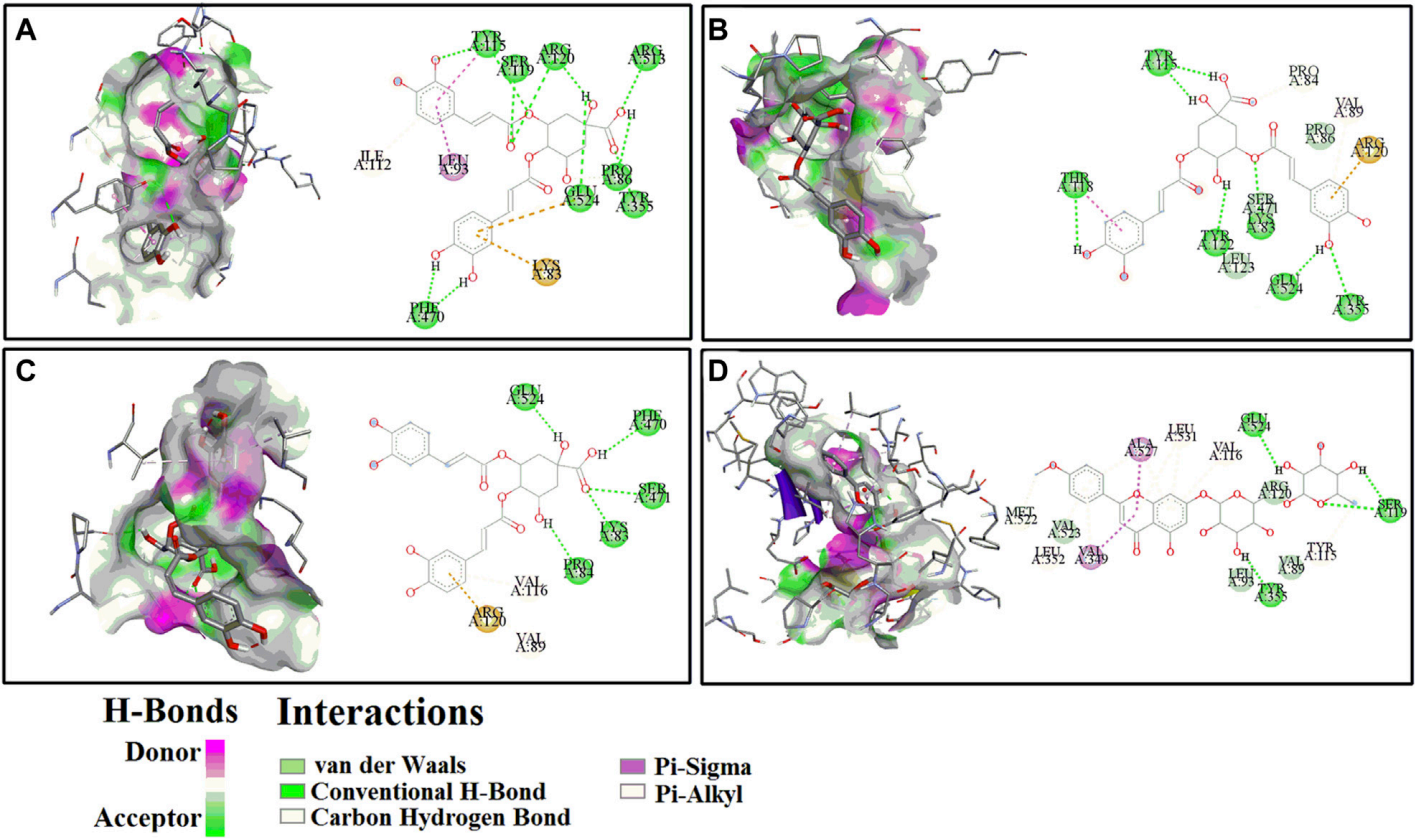
The representative 3D (left) and 2D (right) diagrams of receptor-ligand interactions between COX-2 and peaks 8 **(A)**, 9 **(B)**, 10 **(C)** and 12 **(D)**, respectively.

Presently, the molecular docking analysis was used to simulate the interactions between HAase, COX-2 and the potential ligands in CIFE. The binding energy (BE), inhibition constant (Ki), and the key amino acids involved in hydrogen bonds (H-bond) were listed in Table 3. The 3D and 2D docking diagrams between peaks 8–10, 12 and COX-2 were depicted in Figure 4. Thereinto, aspirin (Asp)/indomethacin (Indo) and ascorbic acid were employed to be positive controls for COX-2 and HAase, respectively.

**TABLE 3:** The docking results of the potential ligands in CIFE against COX-2 and HAase.

Peak no.	COX-2			HAase		
	BE (Kcal/mol)	Ki (µM)	H-Bonds	BE (Kcal/mol)	Ki (µM)	H-Bonds
1	−6.97	7.82	Met522, Ala527	−6.81	10.13	Arg47, Ser303, Ser304, Asp305
2	−7.34	3.58	Pro84, Ser119, Arg120, Glu524	−7.15	5.74	Asp111, Glu113, Tyr184
3	−7.68	2.35	Lys83, Tyr122, Tyr355, Glu524	−6.43	19.46	Tyr55, Glu113, Gln271, Arg274, Trp301
4	−6.95	8.06	Lys83, Ser119, Arg120, Tyr355, Glu524	−5.86	50.57	Asp56, Asp111, Glu113, Tyr184, Ser304
5	−5.75	60.87	Val523	−5.77	59.09	Asp56, Arg74, Gln98, Asp111, Glu113
6	−6.64	13.56	Lys83, Pro84, Pro86, Tyr115, Tyr122	−5.34	121.53	Glu113, Ser304
7	−6.32	23.13	Lys83, Ser119, Arg120, Glu524	−5.88	49.05	Asp111, Glu113, Tyr184
8	−7.98	1.42	Pro86, Tyr115, Ser119, Arg120, Tyr355, Phe470, Arg513, Glu524	−6.37	21.32	Tyr55, Asp111, Glu113, Tyr184, Gly302, Ser304
9	−7.63	2.55	Lys83, Tyr115, Thr118, Tyr122, Tyr355, Glu524	−6.69	12.40	Tyr55, Gly58, Gly302, Ser304
10	−7.76	2.05	Lys83, Pro84, Phe470, Ser471, Glu524	−6.35	22.28	Tyr55, Tyr227, Asp111, Arg274, Ser304, Asp305
11	−6.60	14.48	Tyr115, Arg120, Arg513, Glu524	−5.25	142.28	Arg274, Ser303, Asp305
12	−8.29	0.83	Ser119, Tyr355, Glu524	−5.92	46.06	Asp111, Tyr301, Gly302, Ser303
13	−5.11	81.06	Arg120, Tyr355, Met522, Ser530	−5.15	168.0	Glu113, Tyr184, Ser304
Asp	−5.82	53.86	Arg120, Tyr122			
Indo	−9.18	0.19	Val116, Arg120			
AA				−6.1	33.23	Tyr55, Asp111, Glu113, Tyr184

BE, binding energy; H-bond, hydrogen bond; Asp, aspirin; Indo, indomethacin; AA, ascorbic acid; HAase, hyaluronidase; Ki, inhibition constant; Ala, Alanine; Arg, Arginine; Asp, Aspartic acid; Gln, Glutarnine; Glu, Glutamic acid; Lys, Lysine; Met, Methionine; Phe, Phenylalanine, Pro, Proline; Ser, Serine; Thr, Threonine; Tyr, Tyrosine; Val, Valine.

For the COX-2, peak 12 (Linarin) displayed the strongest binding affinity with the lowest BE and Ki value of −8.29 kcal/mol and 0.83 µM, respectively, which were slightly weaker than the positive control Indo (BE: −9.18 kcal/mol, Ki: 0.19 µM) but markedly higher than the other positive control Asp (BE: −5.82, Ki: 53.86 µM). Similarly, 4 chlorogenic acid isomers, including peak 8 (BE: −7.98 kcal/mol, Ki: 1.42 µM), peak 9 (BE: −7.63 kcal/mol, Ki: 2.55 µM), peak 10 (BE: −7.76 kcal/mol, Ki: 2.05 µM), peak 2 (BE: −7.34 kcal/mol, Ki: 3.58 µM), peak 1 (BE: −6.97 kcal/mol, Ki: 7.82 µM), and peak 3 (BE: −7.68 kcal/mol, Ki: 3.35 µM), also exhibited higher binding affinities to COX-2 than the positive control Asp. With respect to HAase, the peak 2 exerted lower BE (−7.15 kcal/mol) and Ki (5.74 µM) values, and it was comparable with the positive control ascorbic acid (BE: −6.10 kcal/mol, Ki: 33.23 µM). Intriguingly, the other chlorogenic acid isomers, such as peak 1 (BE: −6.81 kcal/mol, Ki: 10.13 µM), peak 8 (BE: −6.37 kcal/mol, Ki: 21.32 µM), peak 9 (BE: −6.69 kcal/mol, Ki: 12.40 µM), peak 10 (BE: −6.35 kcal/mol, Ki: 22.28 µM), and peak 3 (BE: −6.43 kcal/mol, Ki: 19.46 µM), also displayed higher binding strength to HAase.

Meanwhile, for the specific interaction sites, the aforementioned ligands fished out from CIFE formed diverse H-bonds with their correlated target enzymes. For instance, linarin (peak 12, Figure 4D) formed 4 H-bonds with the amino acid residues of COX-2 in the 3D diagram, including Ser119 (2×), Tyr355 and Glu524. More details were displayed in Table 3 and Figure 4. Furthermore, other binding affinity forces, such the as van der Waals, carbon hydrogen bond, Pi-sigma, and Pi-alkyl, were also contributed to the interactions between the linarin and COX-2 complex.

## 4 Discussion

Due to environmental pollution, social pressure, and personal factors, the incidence of sensitive skin (SS) is increasing, people are becoming more conscious of their skin problems. Generally speaking, the occurrence of SS involves multiple levels, among which impaired skin barrier function is the main cause. Damaged skin barrier function will cause a series of reactions in the body (such as immune inflammation), leading to subjective symptoms such as burning, tingling, itching, and redness.

The impaired skin barrier function has been speculated to be closely related to reactive oxygen species in the body (Diao et al., 2021). As the largest organ in humans, the skin is always exposed to air and sunlight, in a state of oxidative stress. Excessive free radicals will attack cell membranes, proteins and even DNA, resulting in degradation of cell structure and function. Therefore, removing excess free radicals in the body helps cell membranes resist free radical damage, thereby maintaining the skin barrier structure and its normal function (Fan et al., 2016). In this study, electron transfer based DPPH and ABTS free radical scavenging assays were employed to asses the antioxidant capacities of CIFE. Our results in Table 1 indicated that CIFE displayed strong scavenging potential on DPPH and ABTS radicals, especially no significant difference with the positive control Trolox (*p* > 0.05), with the IC50 values of 103.47 ± 4.83 μg/mL and 56.25 ± 1.82 μg/mL, respectively. Coincidentally, another work also reported that CIFE prevented DNA damage caused by free radicals, thus possessing the potential as a natural antioxidant (Debnath et al., 2013).

Inflammation is the defensive response of the body to adverse stimulus (Lin et al., 2017; Chen G. L. et al., 2019). Thereof, TNF-α is an important early inflammatory factor that stimulates the activation of IL-6 and IL-1β. Excessive IL-6 often causes inflammatory damage, and elevated IL-6 levels are associated with a variety of inflammatory related diseases, while IL-1β is an endothelial cell activating factor that often acts synergistically with TNF-α (Saiki et al., 2018).

Presently, the results in Figure 1 indicated that CIFE at 5, 10, and 20 μg/mL markedly decreased the levels of pro-inflammatory cytokines in a dose-dependent manner, including IL-1β, IL-6, PEG2, TNF-α, IFN-γ and NO, compared to the Neg group (*p* < 0.01). In particular, CIFE at 20 μg/mL exhibited comparably anti-inflammatory potential with the positive control of aspirin at 10 μg/mL (*p* > 0.05), thus further confirming its clear anti-inflammatory activity. Similarly, another study also found that the supercritical CO_2_ extract of CIF reduced the inflammatory indicators such as TNF-α, IL-1β, IL-6, PGE2, NO, COX-2 and inducible nitric oxide synthase (iNOS) in tissues. In addition, its chemical components, including chlorogenic acid, luteolin-7-glucoside, montmorillonite, luteolin, and leucocephalin, were analysed by gas chromatography-mass spectrometry (GC-MS) and HPLC (Wu et al., 2013). CIF has been commonly used as both food and medicine worldwide, and its water extract and volatile oil are raw materials for various pharmaceutical, chemical, and health products, with good development and application prospects. To this regard, this study explored the varieties of CIFE on the inflammatory factors in LPS induced RAW264.7 cells, providing great significance to interpret the anti-inflammatory mechanism of CIFE in SS disease and develop natural anti-inflammatory drugs.

Arachidonic acid (AA) metabolism is one of the closely related pathways involved in inflammatory response. Under the stimulation of inflammation, AA is catalyzed by COX-2 to produce prostaglandins (PGs), which have physiological activities such as dilating blood vessels, causing pain, and finally causes edema, local erythema and other inflammatory reactions (Chen et al., 2021). COX-2 is mainly expressed under pathological conditions, therefore inhibiting its activity will reduce PGs synthesis and have anti-inflammatory effects (Yao and Narumiya, 2019). HA is a kind of high molecular weight polysaccharide with high hydrophilicity, which has the characteristics of maintaining skin moisture and elasticity, promoting wound healing, and regulating blood vessel formation. At the same time, HA also participates in regulating inflammation and allergic reactions, thus having a good improvement effect on sensitive skin (Voigt and Driver, 2012). HAase is a specific lyase of HA. Inhibition of HAase will alleviate allergic reactions by maintaining the function of HA, thus playing a role in maintaining skin barrier maintenance (Marinho et al., 2021).

As shown in Table 1, CIFE displayed noteworthy inhibition on COX-2 *in vitro* with the IC50 value of 1.06 ± 0.01 μg/mL, which was marvelously lower than the positive control of aspirin of 6.33 ± 0.05 μg/mL (*p* < 0.01), and comparable with indometacin of 0.60 ± 0.03 μg/mL (*p* > 0.05). Also, the CIFE exhibited comparable inhibitory effect against HAase (IC50, 12.22 ± 0.39 μg/mL), with the positive control ascorbic acid (11.03 ± 0.41 μg/mL) (*p* > 0.05). After that, the inhibition type of CIFE on COX-2 and HAase was further investigated. As displayed in Figures 2A,B, all the lines passed through the starting point, and the slopes of the lines decreased with the increase of CIFE concentration, indicating that CIFE was the reversible COX-2 and HAase inhibitors. Meanwhile, the Lineweaver-Burk curves of CIFE on COX-2 and HAase demonstrated a set of straight lines that intersected in the second quadrant, thereinto the Vm and Km gradually decreased and increased, respectively, with the increase of CIFE concentration. Thus, CIFE exhibited a mixed inhibition characteristics of competitive and non-competitive inhibition on COX-2 and HAase. To this end, it was speculated that by inhibiting COX-2 and HAase activities, CIFE might not only alleviate inflammation, but also repair the damaged skin barrier function caused by SS.

Bio-affinity ultrafiltration liquid chromatography-mass spectrometry (UF-LC/MS) is a fast, simple, and effective method for discovering small molecule drugs, which simulates the specific binding between ligands and receptors to quickly screen bioactive compounds, and then utilizes LC-MS to identify those structures. Therefore, this method integrates active components screening and structural identification, and is especially suitable for fishing out potential bioactive compounds from complex natural products mixtures (Chen et al., 2018). As a commonly used both food and medicinal plant worldwide, previous studies have shown that CIF not only exhibited notably anti-inflammatory effect (Matsuda et al., 2002; Cheon et al., 2009; Park et al., 2012), but also displayed good improvements on skin inflammation (Xiong et al., 2021). In this study, the UF-LC/MS method was employed to further screen the potential COX-2 and HAase ligands in CIFE. Figure 3 depicted the HPLC chromatogram profiles of CIFE after affinity ultrafiltration screening, in which 13 peaks exerted various binding affinities to the COX-2 and HAase. According to Table 2, peak 12 with the highest RBA value of 3.43, followed by peaks 8, 10, 9, 3, 2, 1, 4 and 7 were considered as the potential COX-2 ligands, while peaks 1, 2, 3, 8, 10, 9, 12, 7, 5, 4 and 6 were deemed to be the potential HAase ligands, respectively. Furthermore, the diverse RBA values might be likely due to the competitive interactions between the aforementioned potential ligands and COX-2/HAase enzymes in complex CIFE system. In particular, based on the relative binding strength of the RBA values, linarin and several phenolic acids, including isochlorogenic acid A-C, chlorogenic acid, neochlorogenic acid, and 1,3-dicaffeoylquinic acid were preliminary suggested to be the most potential COX-2 and HAase ligands in CIFE.

Studies have shown that phenolic acids exhibited outstanding inhibitory effects on HAase. For example, the *in vitro* HAase inhibition of ethanol extracts from 6 varieties of sorghum bran was detected by quaternary ammonium salt precipitation method. It was found that the ethanol extract of sapwood sorghum bran exhibited the strongest inhibition on HAase, and the inhibitory rate was positively correlated with the total phenol content and iron-reducing antioxidant capacity of each extract (Bralley et al., 2008).

Molecular docking is a widely used computer aided drug design technique to evaluate the interactions between enzymes and their potential ligands, which could comprehensively interpret the possible interaction modes by determining the docking energies, active binding sites and the key residues of receptors (Chen et al., 2020). In this study, the molecular docking analysis was implemented to similated the interactions between COX-2/HAase and their ligands fished out from CIFE, and further explore the underlying mechanisms of action. According to Table 3, 13 components displayed diverse binding affinities to COX-2 and HAase with differentiated BE and Ki values. Interestingly, the lower the BE and Ki values these components exhibited, the stronger binding strength between the ligand-enzyme complexes were. Namely, the trends of BE value and Ki values of the potential ligands in Table 3 fished out from CIFE was consistent with that of RBA values in Table 2. More specifically, linarin and several phenolic acids (isochlorogenic acid A-C, chlorogenic acid, neochlorogenic acid, and 1,3-dicaffeoylquinic acid) possessed higher RBA values in Table 2, along with the lower BE value and Ki values in Table 3. At the same time, Figure 4 displayed the 3D and 2D diagrams of 4 representative receptor-ligand interactions between COX-2 and peaks 8 (isochlorogenic acid B), 9 (Isochlorogenic acid A), 10 (Isochlorogenic acid C) and 12 (linarin), respectively.

Linarin is a kind of Flavonoids, and considered as the marker for quality analysis of *Chrysanthemi indici* Flos in Chinese Pharmacopoeia (2020). For the anti-inflammatory activities, it was found that linarin competitively bond to MD2, inhibited the formation of TLR4/MD-2 dimer, downregulated the increase of TRAF-6, IRAK-1 and MyD88, reduced the nuclear transfer of NF-κB (P65), TNF-α, IL-6, NO, PGE2, and decreased the synthesis of COX-2 and iNOS, suggesting that linarin might reduce the inflammation of chondrocytes by occupying the binding site of MD2 to improve the progression of arthritis in mice (Qi, 2021). In addition, it was found that linarin inhibited the increase of TNF-α, IL-1β, IL-6 and other inflammatory factors in LPS induced RAW264.7 cells (Kim et al., 2016). Furthermore, network pharmacology and *in vitro* experiments found that flavonoids and their glycosides, including linarin, luteolin-7-*O*-β-D-glucoside, apigenin-7-*O*-β-D-glucoside, phenolic acids such as caffeic acid, chlorogenic acid, 3, 4-dicaffeoyl quinic acid, 3, 5-dicaffeoyl quinic acid and others, inhibited the elevated inflammatory mediators of NO, TNF-α, IL-6 and PGE2 in LPS induced RAW264.7 cells, comprehensively expounded the anti-inflammatory activity of flavonoids and phenolic acids from *Chrysanthemi indici* Flos (Tian et al., 2020).

## 5 Conculsion

The present study indicated the prominent anti-inflammatory activities of CIFE. On the one hand, CIFE strongly scavenged the DPPH and ABTS radicals, and significantly reduced the pro-inflammatory cytokines of IL-1β, IL-6, PEG2, TNF-α, IFN-γ and NO in LPS induced RAW264.7 cells in a dose-dependent manner. On the other hand, CIFE reversibly inhibited the COX-2 and HAase enzymes, with a mixed characteristics of competitive and non-competitive inhibition type. Furthermore, the potential COX-2 and HAase ligands were quickly fished out with the UF-LC/MS method. Together with the comformation of molecular docking simulations, flavonoids, phenolic acids and their glycosides, especially the linarin and several chlorogenic acid isomers, were considered to be the main potential bioactive components for the anti-inflammatory and SS improvement activities of CIFE. In the following work, we will conduct *in vitro* and *in vivo* anti-SS pharmacological validation of the aforementioned potential ligands in CIFE and elucidate their mechanisms of action, providing solid reference for its clinical application. Consequently, the current findings provided further insights of CIF on the amelioration of SS, which might facilitate this valuable both food and medicinal plant to be a potential anti-SS agent.

## Data Availability

The raw data supporting the conclusion of this article will be made available by the authors, without undue reservation.
